# Efficient Removal of Dental Plaque Biofilm from Training Typodont Teeth via Water Flosser

**DOI:** 10.3390/bioengineering10091061

**Published:** 2023-09-08

**Authors:** Yue Wang, Hongyu Gao, Lili Chang, Jingchen Xu, Xueer Zhou, Chaoliang Zhang, Qiang Peng

**Affiliations:** State Key Laboratory of Oral Diseases, National Clinical Research Center for Oral Diseases, West China Hospital of Stomatology, Sichuan University, Chengdu 610041, China

**Keywords:** antibacterial, dental caries, water flosser, oral diseases, biofilms

## Abstract

Plaque biofilms play critical roles in the development of dental caries. Mechanical plaque control methods are considered to be most effective for plaque removal, such as brushing teeth or using flosser. Recently, water flosser has been paid much attention. Here, we tested the ability of a water flosser to remove the adhered sucrose and the dental plaque biofilms formed by *Streptococcus mutans*, *Streptococcus sanguinis*, and *Actinobacillus viscosus*. We found that the residual sucrose concentration was 3.54 mg/mL in the control group, 1.75 mg/mL in the syringe group (simulating the ordinary mouthwash), and 0 mg/mL in water flosser group. In addition, the residual bacterial concentration was 3.6 × 10^8^ CFU/mL in the control group, 1.6 × 10^7^ CFU/mL in the syringe group, and only 5.5 × 10^5^ CFU/mL in the water flosser group. In summary, water flosser is effective for cleaning the teeth, which may have significant potential in preventing dental caries and maintaining oral health.

## 1. Introduction

Dental caries is one of the most prevalent chronic infection diseases in the world, which is characterized by the demineralization and destruction of dental hard tissue [1,2,3]. In addition to the influence on oral health, dental caries and its complications can also induce or aggravate systemic diseases such as diabetes and cardiovascular disease [4,5], severely reducing the quality of human life and causing a huge economic burden. The global prevalence of dental caries is expected to increase in the coming years due to the increased intake of sugary foods in children and adults and the lack of concomitant improvement in oral cleaning efficiency [6].

The acid-producing metabolic activity in dental plaque is the direct cause of caries. Bacteria metabolize carbohydrates to produce lactic acid and other organic acids, which can cause enamel dissolution, mineral loss, and caries formation [7,8]. In addition, low pH conditions resulting from carbohydrate metabolism lead to the increase in acidogenic bacteria in dental plaque [9]. Sucrose is considered to be the most cariogenic carbohydrate [10]. Oral biofilm is formed by the adsorption of salivary protein or glycoprotein to the tooth surface, which is the important foundation of, and internal environment for, the formation and development of dental plaque [11,12]. Oral biofilms can attach to different types of surfaces in the oral cavity, even prosthetic devices and dental implants [13]. *Streptococcus mutans* (*S. mutans*), a recognized caries-causing bacterium, is considered to be an important pathogenic factor in the development of caries [14,15]. It has been shown that sucrose increases the proportion of *S. mutans* in oral biofilms, which may shift the biofilm to a more cariogenic one [16]. As the resident bacteria in the oral cavity, *Streptococcus sanguis* (*S. sanguis*) is the dominant bacterium for early colonization of the tooth surface and accounts for the largest proportion of oral flora [17,18]. *Actinomyces viscosus* (*A. viscosus*) is colonized on the tooth surface or in the periodontal pocket, providing attachment conditions for other pathogenic bacteria [19,20]. More importantly, *A. viscosus* has a strong role in stimulating the inflammatory response [21]. It is found that *A. viscosus* can be isolated from carious lesions on the root surface of human teeth, accounting for its considerable proportion in biofilms [22]. Therefore, removing dental biofilms via appropriate methods is a crucial step in the prevention of caries.

Currently, popular oral plaque control measures can be divided into mechanical and chemical methods. Mechanical plaque control methods mainly include brushing and the use of adjacent surfaces cleaning tools [23]. Chemical plaque control methods mainly include the use of chemical agents such as toothpaste and mouthwash [24,25]. Among them, mechanical plaque control methods are regarded as the most effective methods by which to remove plaque and prevent dental caries. It has been shown that using the Bass method to brush in the morning and evening can effectively reduce the accumulation of plaque on the tooth surface, but about 40% of the tooth surface cannot be cleaned thoroughly [26]. In addition, plaque still remains after using dental floss to clean the uneven adjacent tooth surface.

The water flosser is a new type of oral hygiene maintenance device which uses pulsed water flow to clean the tooth surface and interdental spaces. The water flosser can mix air bubbles in the water flow to produce a vibration-like impact [27,28]. However, the effect of water flosser on the removal of dental plaque from the tooth surface remains controversial.

**Figure 1:**
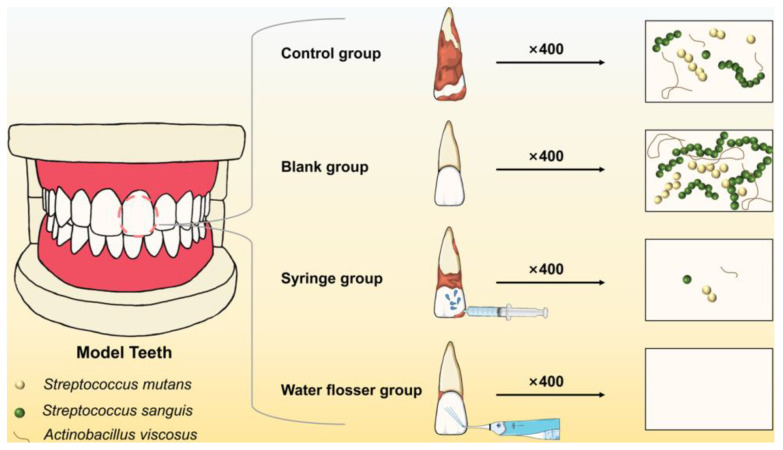
Schematic illustration of removing the dental plaque biofilm from the training typodont model via syringe washing and water flosser.

In this study, three bacterial strains closely related to caries (*S. mutans*, *S. sanguis*, and *A. viscosus*) were chosen to construct biofilm models. Syringe rinsing was used to simulate ordinary mouthwash. The role of water flosser in oral hygiene maintenance was clarified by comparing the difference in the effects of water flosser and mouthwash on sucrose removal and plaque control on the smooth surface of resin teeth. It is hypothesized that water flosser can achieve the better removal of sucrose and dental plaque biofilm than ordinary mouthwash due to its strong and pulsed water flow and thus provide a better guide for patients in oral hygiene maintenance (Figure 1).

## 2. Materials and Methods

### 2.1. Materials

*Streptococcus mutans* UA159 (*S. mutans*), *Streptococcus sanguis* (*S. sanguis*), and *Actinomyces viscosus* (*A. viscosus*) were obtained from the American Type Culture Collection (ATCC, Manassas, VA, USA). Training typodonts and typodont teeth were purchased from Liyue Model Technology Co., Ltd. (Dongguan, China). Brain–heart infusion (BHI) broth was purchased from Oxoid (Basingstoke, England). Sucrose, yeast extract, crystal violet, and the sucrose content test kit were purchased from Solarbio (Beijing, China). All other chemicals used were of analytical grade or better. The Beizhi P50 water flosser was provided by Shanghai Feixiang Technology Co., Ltd. (Shanghai, China).

### 2.2. Removal of Adhered Sucrose Using Water Flosser and Syringe

The sucrose adhesion to typodont teeth was established by immersing typodont teeth in 10% (*w*/*v*) sucrose solution for 1 h followed by air-drying. The adhered sucrose was examined via SEM (scanning electron microscopy) and quantified with the sucrose content test kit according to the manufacturer’s instructions. In the sucrose removal experiment, the sucrose-adhered teeth were divided into a control group, a syringe group, and a water flosser group. In the control group, the teeth were not treated. In the syringe group, the labial surface of the teeth was washed with syringe water flow for 10 s to simulate the general mouth-rinse. In the water flosser group, the labial surface of the teeth was washed with water stream for 10 s. Pristine teeth were used as the blank group. The residual sucrose on the teeth was also examined via SEM and quantified with the sucrose content test kit.

### 2.3. Bacterial Growth Conditions

*A. viscosus* was cultured routinely at 37 °C under anaerobic conditions (10% H_2_, 10% CO_2_ and 80% N_2_) and supplemented with BHI medium containing 5 μg/mL yeast extract. *S. mutans* and *S. sanguis* were cultured in almost the same conditions as above without yeast extract. 

### 2.4. Bacterial Growth Curves in Different Temperature

*S. mutans*, *S. sanguis*, and *A. viscosus* bacterial suspensions were diluted in fresh media to an optical density value corresponding to 10^5^ colony-forming units (CFU)/mL. The above bacteria were cultured under anaerobic conditions at three temperatures (4 °C, room temperature, 37 °C). Optical density values of the bacterial suspension at different time points were recorded, and growth curves were drawn accordingly. 

### 2.5. Biofilm Growth Conditions

*S. mutans*, *S. sanguis*, and *A. viscosus* bacterial suspensions were mixed in equal proportions and supplemented with BHI medium containing 1% sucrose and 0.5% yeast extract to obtain the mixed bacterial suspension (10^8^ CFU/mL). Typodont teeth, pre-sterilized by immersion in 75% alcohol for 24 h, were immersed in the above mixed bacterial suspension. Biofilms were grown on the labial surface of typodont teeth for 48 h at 37 °C under anaerobic conditions in a humidified incubator with media changes performed every 24 h. 

### 2.6. Removal of Biofilms Using Water Flosser and Syringe 

The water flosser and syringe devices were positioned in the same position directly opposite the typodonts. In the water flosser group, biofilms grown on labial surfaces were exposed to the water stream with the water flow perpendicular to the crown for 10 s. In the syringe group, biofilms were removed by the syringe water flow as described above. Biofilms in the control group were not removed. In addition, the teeth without biofilms were defined as the blank group.

### 2.7. Crystal Violet (CV) Staining

CV staining is the most commonly used chemical method for quantitative detection of bacterial biofilm, which can be used in multiple bacterial species [29,30]. The typodont teeth in the above four groups were immersed in 0.5% crystal violet dye for 10 min. The stained teeth were rinsed gently in deionized water to remove excess dye and were photographed to observe the biofilm removal.

### 2.8. Quantitative Assessment of Biofilm Removal 

The effect of biofilm removal was examined via CFU counting assay [31]. The biofilms in the water flosser group, syringe group and control group were mechanically scraped and sonicated into deionized water to obtain bacterial suspensions. The bacterial suspension was diluted with BHI and then inoculated onto BHI agar plates. After incubation for 48 h, bacterial CFU was counted. The effect of biofilm removal was presented as the biofilm removal rate compared to the control (loss of viability = (CFU_control_ − CFU_sample_)/CFU_control_).

### 2.9. Scanning Electron Microscopy (SEM)

The morphology of teeth and biofilms was examined via SEM, which can exhibit the microscopic morphology of bacteria and biofilm clearly [32]. Briefly, after exposure to the water flow, the typodont teeth in the above four groups were rinsed with PBS three times and fixed with 2.5% glutaraldehyde overnight at 4 °C, followed by gradient dehydration with ethanol solutions (50, 70, 95 and 100%) for 10 min. After air-drying, the teeth were used for SEM.

### 2.10. Statistics

The experiments were conducted in triplicate and the data were presented as mean ± standard deviation. One-way analysis of variance was used to compare the difference between groups, which was considered statistically significant if the *p*-value was less than 0.05.

## 3. Results and Discussion

### 3.1. Sucrose Removal

**Figure 2:**
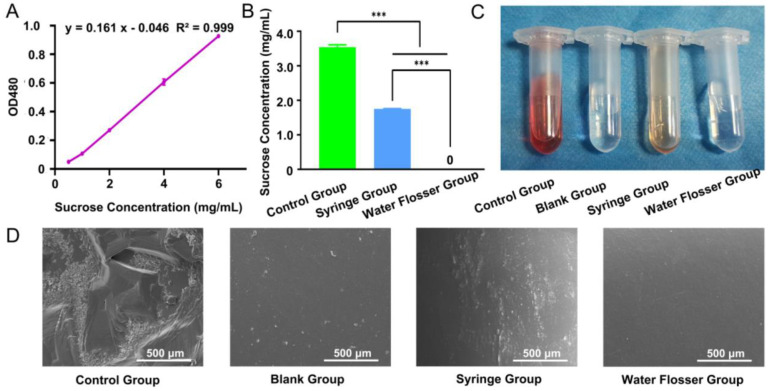
(**A**) Calibration curve for quantification of sucrose concentration. (**B**) Remaining sucrose concentration quantification in the control group, syringe group, and water flosser group. Data are presented as mean ± standard deviation (n = 6). Statistics significance: *** *p* < 0.001. (**C**) Photos of remaining sucrose solutions. (**D**) SEM examination of the morphology of the treated teeth.

The low pH condition resulting from carbohydrate metabolism leads to enamel dissolution, mineral loss, and caries formation [9]. Sucrose is considered the most cariogenic carbohydrate [10]. Therefore, it is important to remove the sucrose on the tooth surface. In our investigation, a standard curve of OD value corresponding to sucrose concentration was plotted (Figure 2A). Then, we further quantified the sucrose removal efficiency via measuring the OD values in the control, syringe, and water flosser groups, comparing them with the standard curve to obtain the concentration of residual sucrose. In the control group, the sucrose concentration was up to 3.54 mg/mL and significantly decreased to 1.75 mg/mL after the use of syringe wash (*p* < 0.001). Residual sucrose concentrations further decreased to 0 using water floss (Figure 2B). Photos of the remaining sucrose solutions in the control, blank, syringe and water floss were taken (Figure 2C). The color of the control group was the darkest, followed by the syringe group. The color of the blank group was nearly transparent, and there was no significant difference between the blank group and the syringe group.

The sucrose removal efficiency of the model teeth was also examined via SEM. We found that there was a large amount of crystalline sucrose remaining on the teeth surface in the control group, while there was no sucrose on the surface of the blank group teeth. After using the syringe, most of the sucrose was removed and only some scattered sucrose particles remained on the teeth surface. Use of the water flosser removed sucrose from the teeth surface to the largest extent, which was almost identical to the blank group (Figure 2D). Compared with the syringe group, the water flosser had the better effect in sucrose removal.

### 3.2. Bacterial Growth Curves 

**Figure 3:**
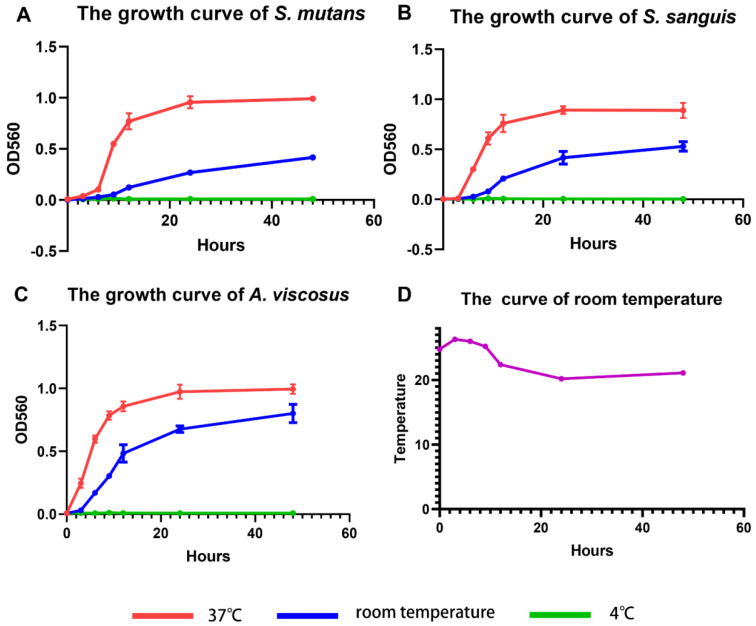
Growth curves of (**A**) *S. mutans*, (**B**) *S. sanguis*, and (**C**) *A. viscosus* at 4 °C, room temperature, and 37 °C. Data are presented as mean ± standard deviation (n = 3). (**D**) Room temperature curve.

The growth of *S. mutans*, *S. sanguis*, and *A. viscosus* at three temperatures was represented via growth curves. The S-shaped curves of *S. mutans* (Figure 3A), *S. sanguis* (Figure 3B), and *A. viscosus* (Figure 3C) at 37 °C were consistent with the traditional sigmoid models, which could be roughly divided into four periods: lag phase; logarithmic phase; stationary phase; decline phase [33]. At room temperature, the growth rate of three bacterial strains slowed down, and the stationary phase did not exhibit at 48 h. At the 48-h time point, the OD value for *S. mutans* (Figure 3A) was approximately 2-fold higher at 37 °C than that at room temperature, while *S. sanguis* (Figure 3B) and *A. viscosus* (Figure 3C) were 1.7 fold and 1.2 fold higher at 37 °C than that at room temperature, respectively. A dramatic reduction in the number of the three bacteria strains was observed at room temperature compared to the same time points at 37 °C. At 4 °C, the growth of the three bacterial strains was significantly inhibited, and the optical density of bacterial suspensions remained essentially unchanged for 48 h (Figure 3A–C). Because room temperature was not constant over 48 h, we monitored room temperature changes during the cultivation of the three bacterial species. The range of room temperature was between 20.2 °C and 26.3 °C, essentially fluctuating around 23 °C (Figure 3D).

Bacterial growth is the coordinated sum of a series of complex processes, including chemical synthesis, assembly, polymerization, biosynthesis, and transport. The activity of bacterial growth is affected by temperature [34,35]. The variation of bacterial growth rate with temperature is a complex process, and the Arrhenius equation suggests that the maximum specific growth rate of bacteria is temperature-dependent [36]. The Verhulst–Pearl model and its variants, such as the Richards model [37], the generalized logistic growth model, the Gompertz model [38], and the Monod bacterial growth model [39], have all been used to describe the growth of bacteria in conditions of dynamic temperature. The growth of the three bacteria stains was temperature-dependent, and it was indicated that 37 °C was close to their optimal growth temperature. Meanwhile, the oral temperature is around 37 °C, so we chose 37 °C as the temperature for subsequent biofilm culture.

### 3.3. General Assessment of Biofilm Removal

**Figure 4:**
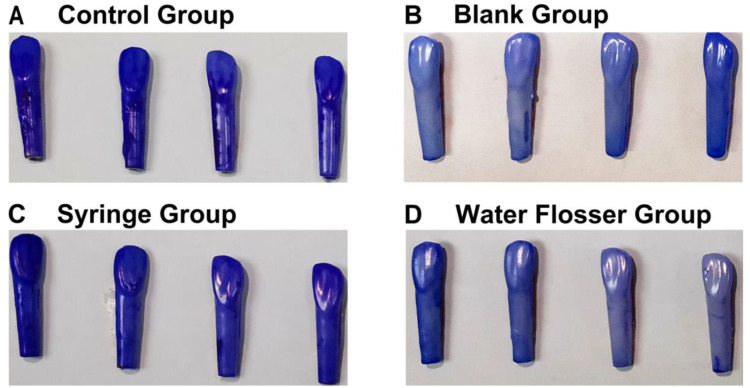
General assessment of biofilm removal: (**A**) the control group; (**B**) the blank group; (**C**) the syringe group; (**D**) the water flosser group.

CV staining to assess total biofilm clearance showed that in the control group, large dark purple plaques almost completely covered the isolated resin teeth, especially the neck and roots of the isolated resin teeth (Figure 4A), while no CV staining was exhibited on teeth in the blank group that had not been immersed in bacterial suspensions of *S. mutans*, *S. sanguis*, and *A. viscosus* (Figure 4B). There was no significant difference between the control group and the syringe group, which still showed large continuous purple staining (Figure 4C). However, almost no CV staining was observed on teeth in the water flosser group, similar to the control group (Figure 4D). This indicated that the water flosser rinsing had a significant effect on biofilm removal and continuity disruption. The significant difference in biofilm removal effects between the two groups can be attributed to the weaker force and larger diameter of the water flow in the syringe group, which caused the water to spread out and have little effect on biofilm removal when it reached the tooth surface. In contrast, the effectiveness of the water flosser is achieved due to the utilization of the fluid shear of high-velocity and ultra-fine water flow.

### 3.4. Quantitative Assessment of Biofilm Removal

**Figure 5:**
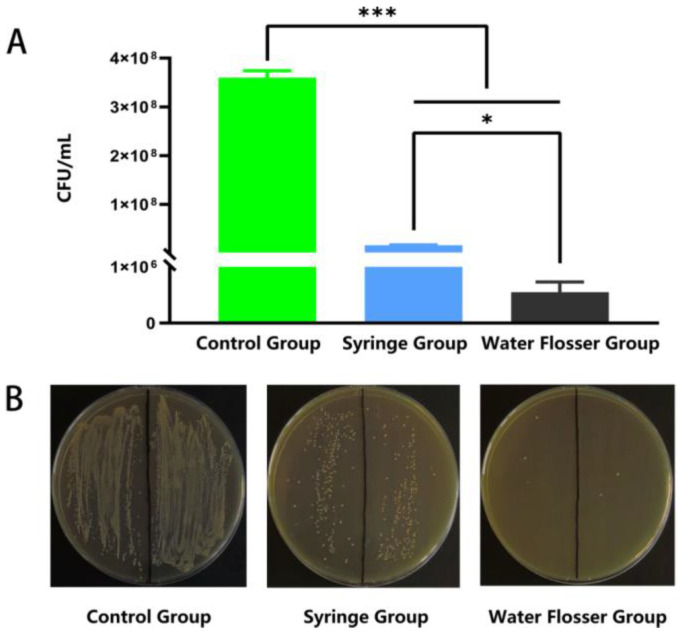
Quantitative assessment of biofilm removal. (**A**) CFU counting-based quantification of the residual bacterial concentration. Data are presented as mean ± standard deviation (n = 6). Statistical significance: * *p* < 0.05, *** *p* < 0.001. (**B**) Typical photos of bacterial colonies grown on BHI agar plates.

We further quantified the biofilm removal efficiency via CFU counting. As shown in Figure 5A, the residual bacterial concentration in the control group was up to 3.6 × 10^8^ CFU/mL and significantly decreased to 1.6 × 10^7^ CFU/mL after the use of syringe wash (*p* < 0.001). The use of water flosser further decreased the residual bacterial concentration to 5.5 × 10^5^ CFU/mL, only 3.4% of that in the syringe group (*p* < 0.05), removing 99.8% of biofilm compared to the control (*p* < 0.001). Figure 5B shows the typical photos of bacterial colonies grown on agar plates with equal dilutions. The number of colonies in the water flosser group is substantially less than those in the control group and the syringe group, which is well-consistent with the quantification results shown in Figure 5A. These results indicated that the water flosser could effectively remove biofilms on the model teeth, and the efficiency was significantly better than that of the syringe. 

### 3.5. SEM Examination

**Figure 6:**
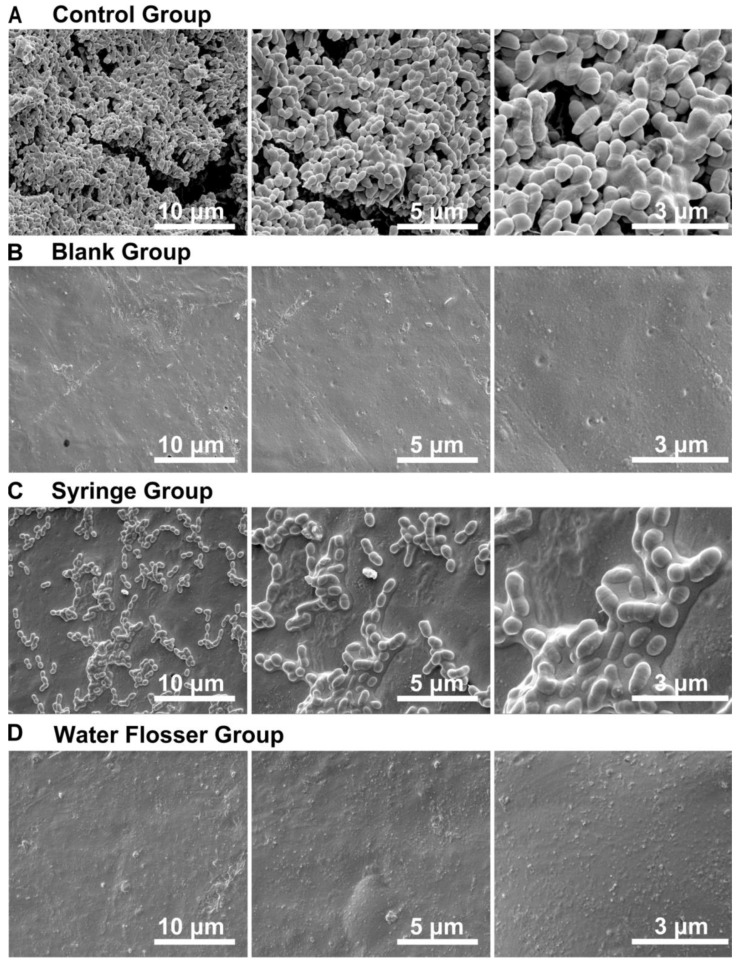
SEM examination of the model teeth surface in (**A**) the control group, (**B**) the blank group, (**C**) the syringe group, and (**D**) the water flosser group.

The biofilm removal efficacy from the model teeth was also examined via SEM. We found that biofilm was well formed in the control group, with a large amount of multilayered bacteria presented on the teeth surface (Figure 6A), while the surface of the blank teeth is quite plain (Figure 6B). Upon the use of syringe wash, the biofilm was substantially broken, and only some scattered bacteria were found on the surface of model teeth (Figure 6C). As expected, use of water flosser removed the biofilm from the teeth surface to the largest extent, and the teeth surface was found to be the same as that in the blank group (Figure 6D). These results are well consistent with those shown in Figure 4 and Figure 5 above and indicate that water flosser is able to efficiently remove the biofilm plaque on the model teeth. 

Effective cleaning was achieved in this study with only pure water from the water flosser at room temperature, without the need for antibiotics or high temperature conditions. This is a great advantage in avoiding the risk of antibiotic resistance and patient health hazards. In addition, the biofilm removal in this study was achieved in a relatively short time period of ten seconds. Good experiences with high cleaning efficiency will facilitate wide applications of the water flosser. In order to maintain the teeth cleaning effect of water flosser, some functional biomaterials with bioadhesive and antibacterial activity may be added. 

## 4. Conclusions

In this work, we found that the growth of three oral pathogens showed significant temperature dependence, with the rate of bacterial growth slowing down as the temperature decreased. Moreover, we compared the efficiency of sucrose and biofilm removal between syringe rising and water flosser. We found that the water flosser is more effective in removing the sucrose and biofilm grown on the surface of model teeth compared to syringe rinsing. The use of water flosser is a potential practical and effective method for oral biofilm removal and improving oral hygiene.

## Data Availability

Data will be made available on request.
